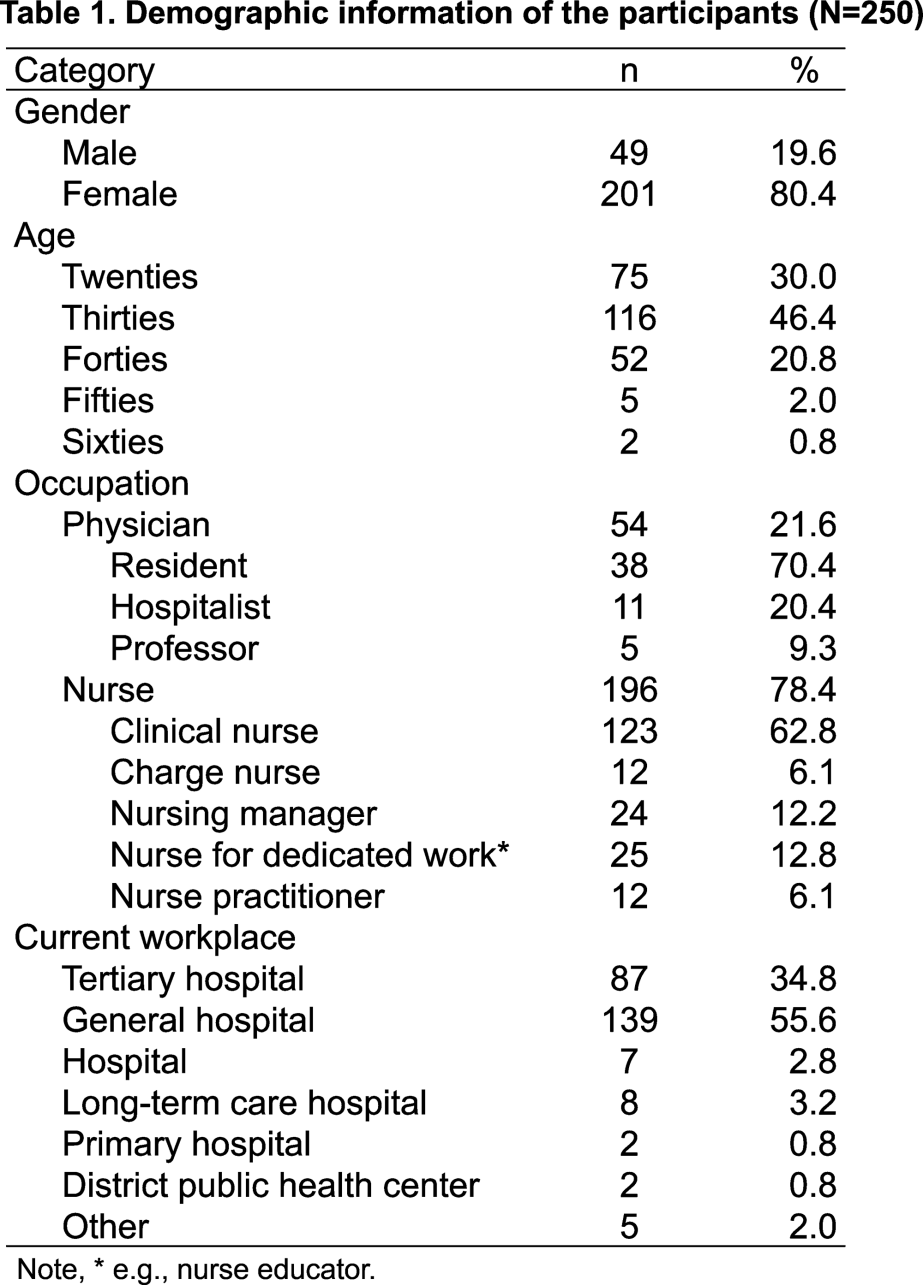# Experience with Using Personal Protective Equipment among Korean Healthcare Personnel: For the Development of Better Products

**DOI:** 10.1017/ash.2024.278

**Published:** 2024-09-16

**Authors:** JaHyun Kang, EunJo Kim

**Affiliations:** Seoul National University College of Nursing; Seoul National University

## Abstract

**Background:** Although various difficulties and self-contamination concerns have been continuously reported regarding the use of personnel protective equipment (PPE) among healthcare personnel (HCP), better PPE options are unavailable in healthcare settings. This study aimed to thoroughly examine HCP’s PPE experience to develop improved PPE. **Method:** By sending cooperation requests to eight academic societies (e.g., Korean Society of Infectious Disease, Korean Society of Critical Care Nursing), four hospitals (e.g., National Medical Center), and Korea Disease Control and Prevention Agency, 250 HCP who had direct patient contact with emerging infectious diseases were recruited. The questionnaire with 65 main questions (211 maximum including sub-questions and 19 open-ended questions) was developed in detail (e.g., use frequency, priority check among the options) based on literature reviews and verified by an expert panel. Through an online survey link, participants completed the questionnaire between December 6-11, 2023, and received a $53 incentive. Descriptive statistics were performed for data analysis. **Results:** Among 250 participants, most were female (80%), in their thirties (46.4), nurses (78%,), working at general hospitals (56%), and averaged 121.1 months of clinical experience (Table 1). Among 6 PPE sets (Figure 1), 29% of participants used Set #2 (i.e., disposable water-proof gown, gloves, goggles/face shield, hair cap, and N95 mask) and 26% used Set #3 (i.e., Level D, gloves, goggles/face shield, shoe cover, N95 mask, and hair cap). 64% of HCP preferred Set #2 due to practical aspects (e.g., simple, convenient, safer than Set #1, and easy donning/doffing). Most PPE sets were largely used for 30 minutes to 1 hour (except level D, to 2 hours). Most prioritized difficulties of PPE use were: disposable mask dampness; pressure pain on the wearing area for N95 masks; powered air purifying respirator (PAPR) wear takes a long time; sweating for gloves, waterproof gowns, and level D/C coveralls; skin exposed through torn gloves; blurry visibility for goggles/face shield; hearing difficulty for hoods; and a slippery bottom for shoe covers. Moments for self-contamination concerns were: adjusting PPE that has slipped down (e.g., masks, gowns, gloves, goggles/face shield, and hoods); doffing hood procedure (e.g., PAPR); and skin contact of shoe cover surfaces during doffing. Most difficulties when using PPE combinations were unfamiliarity with donning/doffing procedures. Participants wish to develop various PPE sizes which can be easily donned/doffed intuitively (e.g., well-ventilated integral PPE). **Conclusion:** Collaboration with manufacturers based on our results is necessary to develop better PPE options for HCP’s safety and satisfaction.

**Figure:**
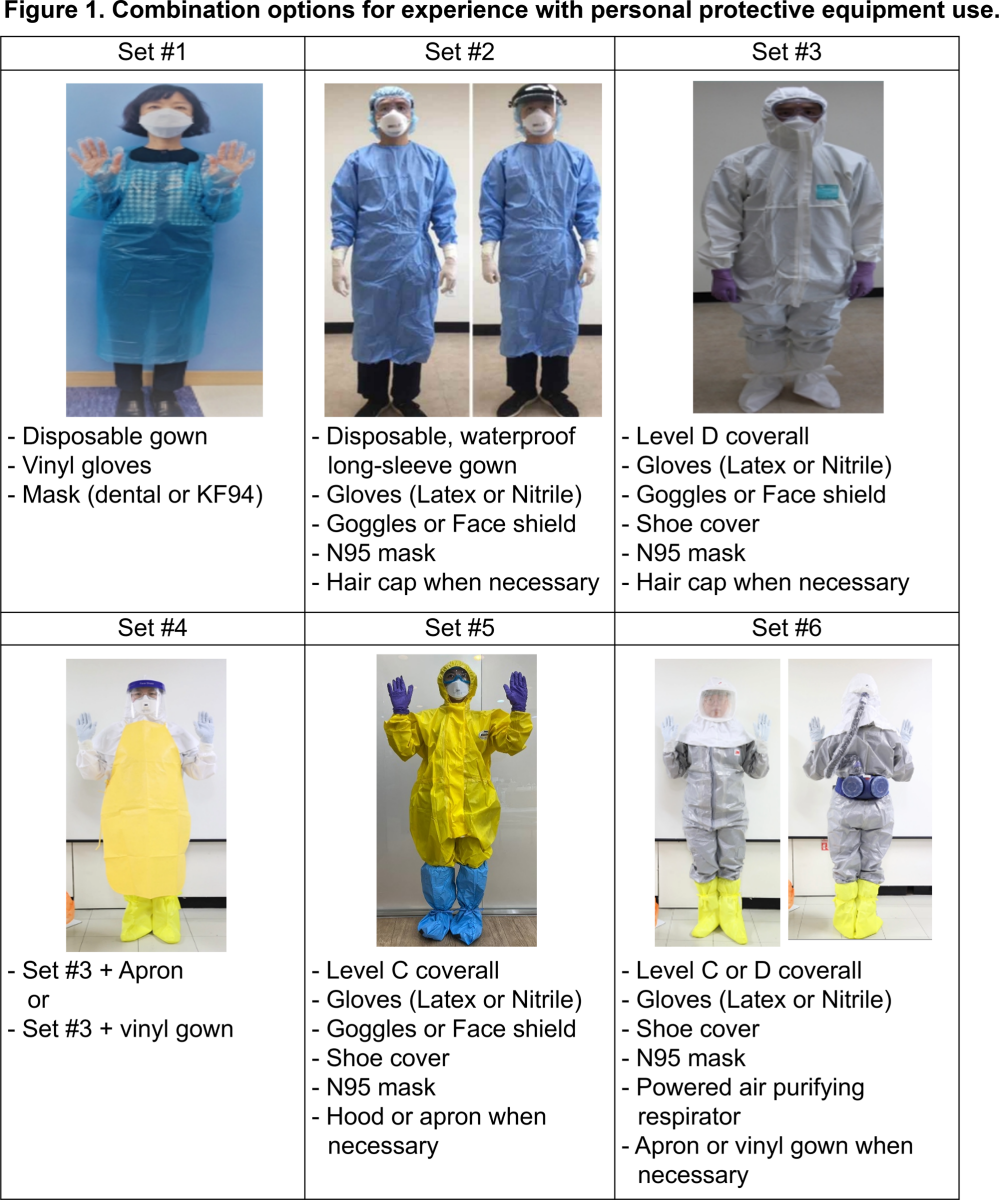

**Figure:**